# An Enzyme That Inactivates the Inflammatory Mediator Leukotriene B_4_ Restricts Mycobacterial Infection

**DOI:** 10.1371/journal.pone.0067828

**Published:** 2013-07-11

**Authors:** David M. Tobin, Francisco J. Roca, John P. Ray, Dennis C. Ko, Lalita Ramakrishnan

**Affiliations:** 1 Department of Molecular Genetics and Microbiology, Duke University Medical Center, Durham, North Carolina, United States of America; 2 Center for Microbial Pathogenesis, Duke University Medical Center, Durham, North Carolina, United States of America; 3 Center for AIDS Research, Duke University Medical Center, Durham, North Carolina, United States of America; 4 Department of Microbiology, University of Washington, Seattle, Washington, United States of America; 5 Department of Medicine, University of Washington, Seattle, Washington, United States of America; 6 Department of Immunology, University of Washington, Seattle, Washington, United States of America; University of Cape Town, South Africa

## Abstract

While tuberculosis susceptibility has historically been ascribed to failed inflammation, it is now known that an excess of leukotriene A_4_ hydrolase (LTA4H), which catalyzes the final step in leukotriene B_4_ (LTB_4_) synthesis, produces a hyperinflammatory state and tuberculosis susceptibility. Here we show that the LTB_4_-inactivating enzyme leukotriene B_4_ dehydrogenase/prostaglandin reductase 1 (LTB4DH/PTGR1) restricts inflammation and independently confers resistance to tuberculous infection. LTB4DH overexpression counters the susceptibility resulting from LTA4H excess while *ltb4dh*-deficient animals can be rescued pharmacologically by LTB_4_ receptor antagonists. These data place LTB4DH as a key modulator of TB susceptibility and suggest new tuberculosis therapeutic strategies.

## Introduction

An effective host immune response balances effective microbial killing mechanisms and damage to the host itself. Recent work has shown dysregulation of these responses in tuberculosis, either by immunodeficiency or by overexuberant immune activation, worsens outcome by increasing bacterial burdens [1], [2]. The balance of pro- and anti-inflammatory eicosanoids is particularly critical in the regulation of the host response to infecting mycobacteria [2], [3]. The enzyme LTA4H, which catalyzes the synthesis of LTB_4_ from its unstable precursor LTA_4_ [4] sits at a key crossroads regulating the balance between the anti-inflammatory lipoxins and pro-inflammatory LTB_4_ [2], [5]. In zebrafish larvae infected with *Mycobacterium marinum*, LTA4H deficiency and excess both produce hypersusceptibility with increased bacterial burdens [2].

The susceptibility of LTA4H deficiency is mediated by the lipoxin excess that results from blocking the enzymatic pathway to LTB_4_ synthesis rather than compromised LTB_4_ production *per se* [2]. In contrast, excess LTB_4_ activity resulting from LTA4H excess plays a critical role in a hyperinflammatory route to increased disease severity [2]. A mechanistic dissection revealed that LTA4H overexpression produces TNF excess during infection and can be rescued by genetic knockdown or pharmacological modulation of TNF [2]. Albeit by a distinct mechanism, LTA4H/TNF excess converges on the same pathway to hypersusceptibility as LTA4H/TNF deficiency: necrosis of infected macrophages that releases the bacteria into the growth-promoting extracellular environment [6]. While LTA4H/TNF deficiency permits uncontrolled intracellular bacterial growth culminating in macrophage lysis, macrophage necrosis occurs in LTA4H/TNF excess despite an initial reduction in intracellular bacterial growth [2].

LTB_4_ is induced in human tuberculosis [7], and the zebrafish findings are corroborated in humans [2]. A human promoter variant that increases LTA4H expression is associated with a similar increase in tuberculosis severity as a low-LTA4H expressing promoter variant. As in zebrafish, the two variants correlate clinically with a high and low inflammatory state, respectively. Importantly, high-activity *LTA4H* genotypes show strong association with a genotype-dependent response to adjunctive anti-inflammatory therapy in TB meningitis. The implication of LTB_4_ as a pharmacologically correctible host susceptibility determinant, led us to investigate whether additional modulators of LTB_4_ might influence tuberculosis susceptibility and provide therapeutic targets for adjunctive therapies.

## Results and Discussion

**Figure 1:**
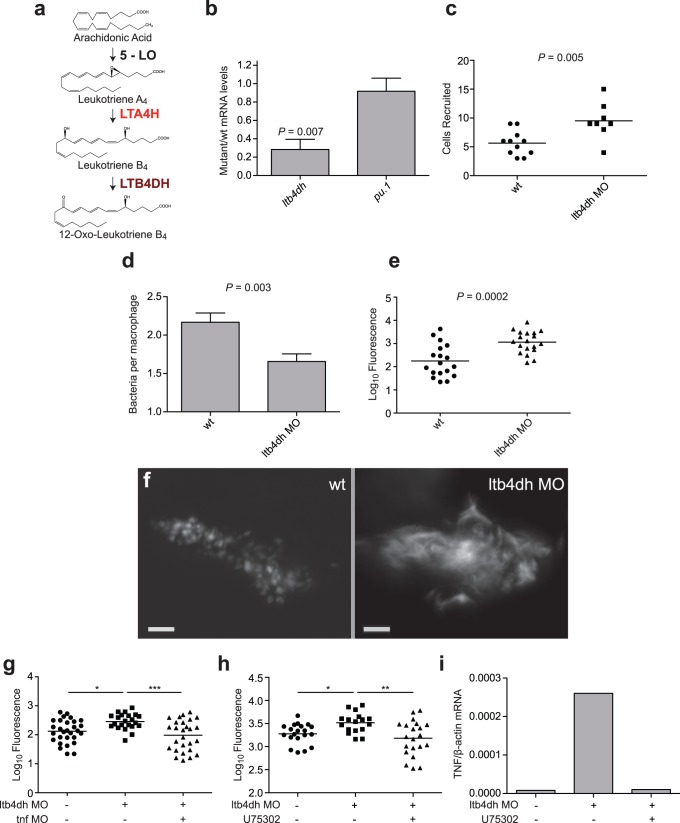
(a) Schematic of LTB4 pathway, with proposed roles for the enzymes LTA4H and LTB4DH in synthesis and inactivation of LTB_4_, respectively highlighted in red. (b) Mean ratio (±SEM) of mutant to wildtype mRNA levels in uninfected 3 dpf *lta4h^zm5961^* animals compared to wildtype animals in four biological replicates. P = 0.007; one sample, two-tailed t test. (c) Number of cells recruited to hindbrain ventricle 6 hpi of sibling wildtype or *ltb4dh* morphant embryos after infection at 24 hpf with 90–100 CFU *M. marinum*. P = 0.005; Students’s unpaired t-test. (d) Mean(±SEM) number of bacteria per infected macrophage in 14 wildtype and 14 *ltb4dh* morphant siblings at 72 hpi after infection with 100–150 *erp* mutant bacteria. P = 0.003; Student’s unpaired t-test. Representative of two independent experiments. (e) Quantitation of bacterial burden by FPC at 5 dpi after infection of sibling controls or *ltb4dh* morphants with 79±21 CFU wildtype *M. marinum*. P = 0.002; Student’s unpaired t-test. Representative of four independent experiments. (f) Representative images of wildtype granuloma and bacterial extracellular cording in *ltb4dh* morphant. Scale bars, 20 µM. (g) Quantitation of bacterial burden by FPC at 3 dpi of sibling controls, ltb4dh morphants and ltb4dh/TNF double morphants with 90±10 CFU wildtype *M. marinum*. Statistical comparisons by one-way ANOVA with Tukey’s post hoc test. (h) Quantitation of bacterial burden by FPC at 4 dpi after infection of sibling controls or *ltb4dh* morphants in vehicle (0.5% DMSO) alone or treated with 10 µM U75302. (i) Relative TNF levels in wildtype siblings or *ltb4dh* morphant larvae 24 hpi with 150–200 CFU *M. marinum* with or without the addition of 10 µM U75302.

**Figure 2:**
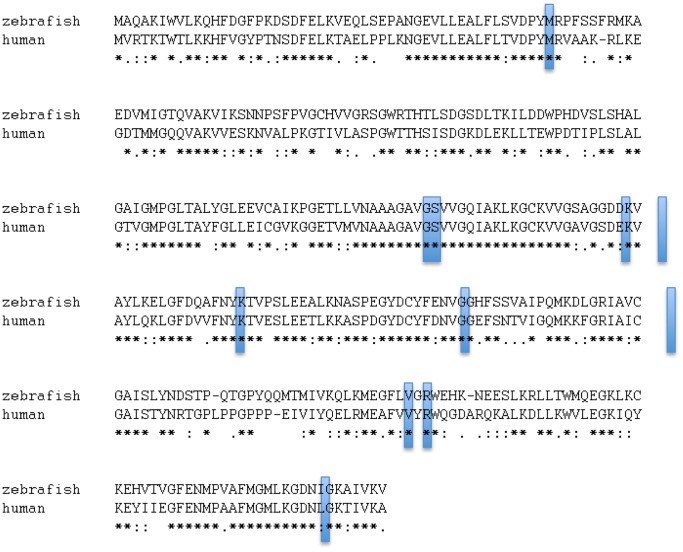
Amino acid alignment of human LTB4DH/PTGR1 with its closest zebrafish orthologue. Identical residues are starred. Residues predicted by crystal structure to interact with NADP+ are shaded with light blue boxes^13^.

We targeted LTB4DH/PTGR1 (henceforth referred to as LTB4DH), an LTB_4_-inactivating enzyme [8], [9] not previously implicated in mycobacterial susceptibility (Fig. 1a). In isolated polymorphonuclear leukocytes, LTB_4_ is inactivated by omega oxidation via P450 while in other tissues via dehydrogenation through LTB4DH; notably the omega oxidation pathway is not predominant in monocytes, suggesting that dehydrogenation may represent the main inactivation pathway in these immune cells most closely associated with tuberculosis [9]–[11]. Consistent with this, LTB4DH/PTGR1 activity is prevalent in human monocytes (as assessed by a second role for the enzyme in lipoxin modification), but this activity is largely absent in human PMNs [12]. The zebrafish orthologue of human *LTB4DH* shares 61% amino acid identity (Fig. 2). All key residues shown to interact with NADP+ in the guinea pig LTB4DH crystal structure are conserved between zebrafish, humans and guinea pigs (Fig. 2) [13]. We found baseline *ltb4dh* RNA levels to be lower in *lta4h* mutant zebrafish than in wildtype siblings (Fig. 1b), suggesting that the *ltb4dh* promoter or transcript stability responds to feedback signals within this pathway. RNA levels of the myeloid transcription factor *pu.1* were not affected by the *lta4h* mutation, providing an internal control (Fig. 1b). Thus, production of *ltb4dh,* the downstream inactivating enzyme, may be limited when its LTB_4_ substrate is not produced due to lack of the upstream enzyme responsible for its generation. This interplay suggests that the two enzymes can function together to regulate LTB_4_.

To determine if LTB4DH functions to limit the inflammatory effects of LTB_4,_ we knocked down *ltb4dh* expression using antisense morpholinos. Even at baseline, *ltb4dh* morphants exhibited 2.9+/−0.4 fold increased *tnf* RNA expression over wildtype siblings (*P = *0.04; three biological replicates). In addition to increased *tnf* expression, LTA4H-overexpressing zebrafish exhibit increased macrophage recruitment to bacteria introduced into the hindbrain ventricle, a compartment normally lacking macrophages [2]. So too did the LTB4DH morphants (Fig. 1c). Thus both the overexpression of the enzyme that produces LTB_4_ and inhibition of its inactivating enzyme result in the signature hyperinflammatory phenotypes attributable to excess levels of LTB_4_. Our *in vivo* findings are consistent with the proposed role of LTB4DH as a negative regulator of LTB_4_ activity [8].

**Figure 3:**
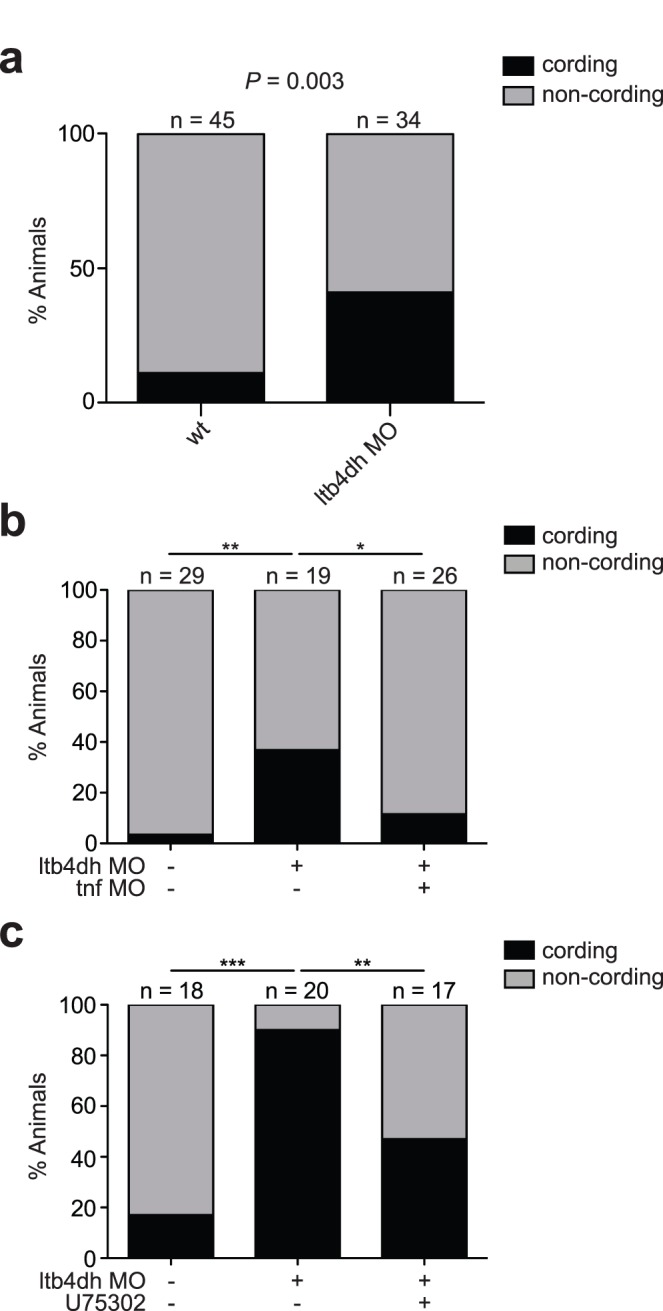
a) Percentage of animals with cording at 4 dpi from 34 *ltb4dh* morphants and 45 wildtype siblings infected with 100–150 wildtype *M.* *marinum*. P = 0.003; Fisher’s exact test of contingency table. b) Percentage of animals in Fig. 1g with cording at 4 dpi among sibling wildtype, *ltb4dh* morphant and *ltb4dh/tnf* double morphant animals. **P = 0.004, *P = 0.043; Fisher’s exact test of contingency table comparing wildtype and *ltb4dh/tnf* double morphant each to *ltb4dh* morphant. c) Percentage of animals in Fig. 1h with cording at 4 dpi among sibling widtype, *ltb4dh* morphants treated with 10 µM U75302 and *ltb4dh* morphants in vehicle (0.5% DMSO). ***P<0.0001, **P = 0.01; Fisher’s exact test of contingency table comparing wildtype and U75302-treated *ltb4dh* morphant each to vehicle-treated *ltb4dh* morphant.

Thus establishing a functional role for LTB4DH in LTB_4_ inactivation, we asked if LTB4DH deficiency produced the same hypersusceptibility phenotype as LTA4H overexpression. *ltb4dh* morphants exhibited the same sequence of phenotypes as LTA4H excess created by mRNA expression: initial decreased intracellular bacterial burden after mycobacterial infection degenerated within a few days into increased bacterial burdens associated with cording morphology characteristic of macrophage lysis and extracellular residence (Fig. 1d–f and Fig. 3a). Morpholino inhibition of TNF in the background of *ltb4dh* morphants restored wildtype infection burden and removed the cording phenotype, showing that TNF is a key mediator of susceptibility as it is with LTA4H excess [2] (Fig. 1g and Fig. 3b).

**Figure 4:**
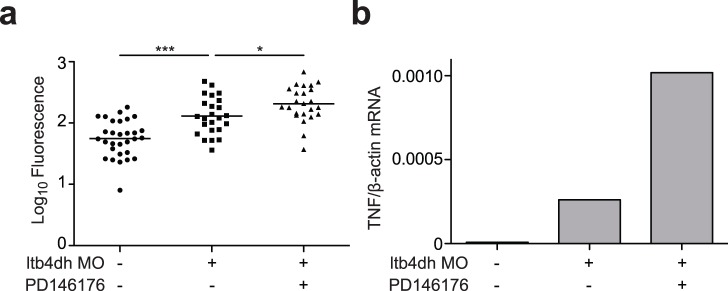
a) Quantitation of bacterial burden by FPC at 3 dpi of sibling controls or *ltb4dh* morphants with 90±10 CFU wildtype *M. marinum* in vehicle (0.5% DMSO) alone or treated with 100 nM PD146176. Statistical comparisons by one-way ANOVA with Tukey’s post hoc test. b) Relative TNF levels in 1 dpi larvae after injection with LTB4DH morpholino and infection with 150–200 CFU *M. marinum* with or without the addition of 500 nM PD146176.

If the hypersusceptibility of *ltbd4h* morphants is due to excess LTB_4_, then it should be possible to rescue this phenotype pharmacologically by blocking LTB_4_ activity. Accordingly, a LTB_4_ receptor antagonist reversed the cording-associated hypersusceptibility of *ltb4dh* morphants while restoring excess *tnf* to wildtype levels (Fig. 1g,h and Fig. 3c). Conversely, interventions that compromise production of anti-inflammatory lipoxins should have a detrimental effect in *ltbd4h* morphants, as they do in states of LTA4H excess [2]. A known 15-lipoxygenase inhibitor produced the expected worsened bacterial burden accompanied by a further increase in *tnf* (Fig. 4). Together these results provide functional evidence that both excess LTA4H and reduced LTB4DH result in increased LTB_4_ activity, which in turn produces mycobacterial hypersusceptibility through TNF-mediated hyperinflammation. These findings suggest that, despite a degree of co-regulation of LTB_4_ production and inactivation, LTB4DH levels can independently modulate control of infection. Importantly, the detrimental effects of LTB4DH deficiency can be countered by pharmacological antagonism of LTB_4_ activity or by directly targeting the resulting excess TNF.

**Figure 5:**
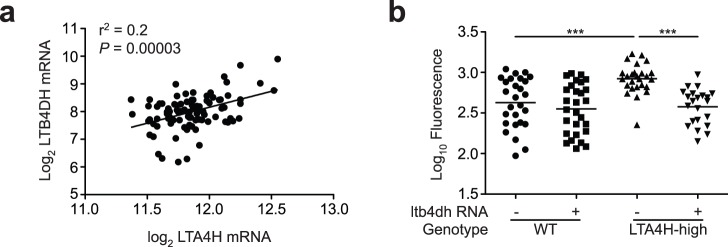
(a) LTA4H and LTB4DH mRNA expression from ^14^ in lymphoblastoid cell lines (LCLs) from Asian cohorts represented in the HapMap project (CHB+JPT). R^2^ = 0.2, P<0.0001; linear regression and F test. (b) Quantitation of bacterial burden by FPC at 3 dpi of sibling controls and LTA4H-high overexpressing simultaneously or not *ltb4dh* with 90±10 CFU wildtype *M. marinum*. Statistical comparisons by one-way ANOVA with Tukey’s post hoc test.

To probe further if LTB4DH can function as an independent resistance factor in humans, we used a dataset from an existing microarray analysis of human HapMap lymphoblastoid cell lines (LCLs) [14] to examine whether the correlation between *lta4h* and *ltb4dh* expression observed in the zebrafish was conserved in humans. We interrogated the collection of Asian cell lines, in which we had previously observed an effect on transcriptional activity of a common *LTA4H* promoter polymorphism [2]. As in the zebrafish, we found a direct correlation between *LTA4H* and *LTB4DH* RNA levels; individual cell lines with higher *LTA4H* transcript levels had higher *LTB4DH* expression (Fig. 5a). The level of correlation (r^2^ of 0.2, *P* = 0.0003) suggests that there is ample space for additional genetic and non-genetic factors to influence LTB4DH expression in humans. Thus, the detrimental effects of LTA4H excess may be dampened only to a limited extent by compensatory induction of the inactivating enzyme, This idea is supported by our zebrafish findings that LTB4DH reduction is an independent and pharmacologically correctible source of hypersusceptibility.

We asked if increasing LTB4DH expression levels *in vivo* might ameliorate the LTA4H-high susceptibility phenotype, thus providing additional potential targets for intervention. Overexpression of *LTB4DH* mRNA was sufficient to rescue the LTA4H-high phenotype (Fig. 5b). In contrast, LTB4DH overexpression did not worsen infection of wildtype animals (Fig. 5b), consistent with previous data where pharmacological inhibition of LTB_4_ receptor did not increase susceptibility [2]. The lack of a protective role for LTB_4_ in tuberculosis is in contrast to the case of other infections where a protective role has been noted for this inflammatory mediator [15]–[17]. In tuberculosis, LTB_4_ may mediate only pathogenesis, as it does in a variety of inflammatory diseases - asthma, atherosclerosis, rheumatoid arthritis, gout, obesity and cancer [18]–[24]. Accordingly, we find that excess LTB_4_ resulting from two independent deviations in its metabolic pathway produces hypersusceptibility to mycobacterial infection.

In conclusion, these findings implicate a downstream inactivating enzyme of a pro-inflammatory eicosanoid as an important controller of mycobacterial resistance. Despite finding some degree of co-regulation of LTB4DH and LTA4H in both zebrafish and humans, *ltb4dh* serves as an independent susceptibility locus in the zebrafish. Similarly, human variation in LTB4DH may influence TB susceptibility. Our work suggests that pharmacological interventions can compensate for the consequences of LTB4DH reduction, providing new approaches to titering eicosanoid balance. LTB_4_-mediated inflammation may have relevance to oncogenic transformation, as LTB_4_ and components of its synthetic pathway are induced in different cancers [23]–[25]. Particularly intriguing from a therapeutic perspective is the ability of gallic acid and a purified compound from *Radix astragali* to induce LTB4DH expression and limit oncogenic transformation [26]. Specific pharmacological inducers of LTB4DH expression, besides serving as novel anti-inflammatory therapies, may provide a new route to addressing the known hypersusceptibility to tuberculosis of individuals with high LTA4H expression [2].

## Materials and Methods

### Bacterial Strains

WT strain M (ATCC #BAA-535) was transformed with plasmids containing transcriptional fusions of the gene encoding Wasabi to a constitutively-expressed Mycobacterium marinum promoter as described [27]. The *erp* mutant expressing *msp-12::gfp* [28] was used for quantitation of intracellular bacterial burdens in Fig. 1d. Bacteria were grown in 7H9 media supplemented with oleic acid-albumin-dextrose-complex and 0.05% Tween-80 unless otherwise stated.

### Zebrafish Strains and Infections

All experiments were conducted in conformity with the Public Health Service Policy on Humane Care and Use of Laboratory Animals using protocols approved by the Institutional Animal Care and Use Committee of the University of Washington. Zebrafish embryos of the AB line were injected with Mm or PBS (mock-injected) using phenol red as a visual marker at 30 hours post fertilization or 48 hours post fertilization (hpf) via caudal vein as described [29]. Hindbrain ventricle injections were performed as described at 24 hpf [30]. Innocula at injection were determined injecting the same volume onto selective bacteriologic plates and enumerating bacteria when colonies formed in 5–7 days. Bacterial burdens of larvae were determined by fluorescence pixel counts (FPC) as described in [27]. After images have been taken with standardized exposure times, FPC counts the number of pixels in each image with values above a background threshold, as determined by matched images of uninfected animals.

### Morpholinos and RNA Injections

Morpholinos were obtained from Genetools (Eugene, OR). Control and *tnf* morpholinos were as previously described [31], [32]. *ltb4dh* morpholino targeted with an injection volume of 2–4 nL of 1 mM morpholino targeting the start codon (sequence 5′ CTTGAGC**CAT**GACTCTTTTTTTGCA 3′, with complement of ATG bolded). All morpholino infections were performed on paired needles; an equal number of morpholino and control animals were injected on each needle in an alternating sequence to reduce variations in dosage between groups.

### RT-PCR

Quantitative RT-PCR was performed as previously described [2], [32]. Total RNA obtained by Trizol extraction from infected or mock-injected embryos were used as templates for generating cDNA (Superscript II reverse transcriptase; random hexamer primers; Invitrogen) for quantitative real-time RT-PCR analysis. qRT-PCR assays were performed such that each 20 µL reaction contained either 250 nM of gene-specific primers or b-actin specific control primers. SYBR green PCR Master Mix (Applied Biosystems) was used at 1X. All qRT-PCR assays were performed in triplicate with an ABI Prism 7300 Real Time PCR System (Applied Biosystems). Data were normalized to b-actin (ΔΔCT analysis). Primer sets used for *ltb4dh* were F 5′ TCTTGGATGACTGGCCTCAT 3′ and R 5′ TCCTGGTTTGATGGCACATA 3′.

*lta4h* and *ltb4dh* RNA was synthesized using the mMessage mMachine kit (Ambion) and the polyA Tailing kit (Ambion). 2–4 nL of RNA was injected at one-four cell stage at a concentration of 200 ng/µL to create LTA4H-high animals and to overexpress *ltb4dh*.

### Pharmacological Interventions in Zebrafish

After infection, small molecules were applied via soaking. Solutions were changed daily. All conditions and controls were standardized to a final concentration of 0.5% DMSO. PD 146176 (BIOMOL) was applied at a final concentration of 500 nM 16 hpi. U75302 (BIOMOL) was applied at a concentration of 10 µM directly after infection.

### Microscopy

Microscopy was performed on a Nikon E600 equipped with DIC optics, a Nikon D-FL-E fluorescence unit with 100 W Mercury lamp and MFC-1000 z-step controller (Applied Scientific Instrumentation) or, for whole animal images, a motorized Nikon inverted Ti-E microscope. Objectives used included 2× Apo Objective 0.1 NA, 10× Plan Fluor 0.3 NA, 40× Plan Fluor 0.75 NA and 60× Oil Plan Apo, 1.4 NA. Widefield fluorescence and DIC images were captured on a CoolSnap HQ or CoolSnap CF CCD camera (Photometrics) using MetaMorph 7.1 (Molecular Devices).

### Statistical Analysis

Statistical analysis was performed with Prism (Graphpad Software) for all comparisons.
